# Lignin nanoparticles as a highly efficient adsorbent for the removal of methylene blue from aqueous media

**DOI:** 10.1038/s41598-024-59612-4

**Published:** 2024-04-19

**Authors:** Reza Pourbaba, Ali Abdulkhani, Alimorad Rashidi, Alireza Ashori

**Affiliations:** 1 Department of Wood and Paper Sciences and Technology, Faculty of Natural Resources, University of Tehran, Karaj, Iran; 2 Nanotechnology Research Center, Research Institute of Petroleum Industry (RIPI), Tehran, Iran; 3 Department of Chemical Technologies, Iranian Research Organization for Science and Technology (IROST), Tehran, Iran

**Keywords:** Lignin nanoparticles, Adsorption, Methylene blue, Hydropic synthesis, Fast absorbent, Environmental sciences, Materials science

## Abstract

This work demonstrated enhanced adsorption capabilities of lignin nanoparticles (LNPs) synthesized via a straightforward hydrotropic method compared to pristine lignin (PL) powder for removing methylene blue dye from aqueous solutions. Kraft lignin was used as a precursor and p-toluenesulfonic acid as the hydrotrope to produce spherical LNPs with ~ 200 nm diameter. Extensive characterization by SEM, AFM, DLS, zeta potential, and BET verified successful fabrication of microporous LNPs with fourfold higher specific surface area (14.9 m^2^/g) compared to PL (3.4 m^2^/g). Significantly reduced particle agglomeration and rearranged surface chemistry (zeta potential of −13.3 mV) arising from the self-assembly of lignin fractions under hydrotropic conditions enabled the application of LNPs and superior adsorbents compared to PL. Batch adsorption experiments exhibited up to 14 times higher methylene blue removal capacity, from 20.74 for PL to 127.91 mg/g for LNPs, and ultrafast equilibrium uptake within 3 min for LNPs compared to 10 min for PL. Kinetic modeling based on pseudo-first-order and pseudo-second-order equations revealed chemisorption as the predominant mechanism, with a rate constant of 0.032825 g/mg·h for LNPs—over an order of magnitude higher than PL (0.07125 g/mg·h). Isotherm modeling indicated Langmuir monolayer adsorption behavior on relatively uniform lignin surface functional groups. The substantially augmented adsorption performance of LNPs arose from the increased surface area and abundance of surface functional groups, providing greater accessibility of chemically active binding sites for rapid dye uptake. Overall, this work demonstrates that tailoring lignin nanoparticle structure and surface chemistry via scalable hydrotropic synthesis is a simple and sustainable approach for producing highly efficient lignin-based nano-adsorbents for organic dye removal from industrial wastewater.

## Introduction

Water pollution caused by industrial activities is a primary environmental concern threatening human health and aquatic ecosystems globally. Industries including textiles, paints, paper, leather, and printing discharge hundreds of different dyes into water resources, with over 70 million tons utilized annually^1^. Approximately 10–15% of these dyes enter the environment as wastewater effluents^2,3^. Even at low concentrations, these colorful compounds noticeably alter the natural appearance of water bodies^4–7^. Beyond aesthetic impacts, dye pollutants have wide-ranging detrimental effects. They lower dissolved oxygen levels in water by inhibiting sunlight penetration, disturbing aquatic life^8^. Many dyes and their degradation products are toxic, carcinogenic, or mutagenic. Contact with contaminated water can cause skin irritations and gastrointestinal disorders in humans^9^.

Among the concerning dye pollutants, methylene blue is one of the most prevalent and hazardous. As a cationic thiazine dye, methylene blue adversely affects aquatic ecosystems by inhibiting photosynthesis and oxygen transport in water^3^. It can also induce eye burns, nausea, vomiting, diarrhea, and tissue necrosis in humans when ingested^8^. Methylene blue is a dark green powder at room temperature, which yields a blue solution when dissolved in water. It is widely used as a dye and stain in textile, pharmaceutical, printing, and other industrial applications^9^. However, the extensive utilization of methylene blue has raised concerns regarding its environmental and health impacts. To mitigate these hazards, effective wastewater treatment methods are needed to remove methylene blue before discharge from industrial facilities. Processes such as ion exchange, oxidation, coagulation, and photodegradation can degrade the dye^10^. However, adsorption has attracted much interest due to its low cost, simplicity, and lack of byproduct generation. In adsorption, molecules are concentrated from a bulk liquid or gas phase onto the surface of a solid adsorbent. The accumulation of adsorbates occurs on the surface due to its higher energy state compared to the bulk material. The lower Gibbs surface free energy is the thermodynamic driving force behind this adsorption process^8^. However, this phenomenon is limited to the exterior of the adsorbent and does not penetrate its internal structure. Identifying the most suitable adsorbents and optimal conditions for efficient removal of methylene blue through adsorption remains an active area of research^9^.

Lignin, an intricate biopolymer found in plants, serves as an abundant and valuable source of aromatic compounds. Regarded as a highly promising natural resource, it holds significant potential for the synthesis of commercially valuable products. In the present day, lignin is primarily obtained as a byproduct from the paper and pulp industry, with a remarkable annual global production that exceeds 170 billion metric tons^11^. This vast availability of lignin presents exciting opportunities for its utilization in the development of innovative applications and sustainable technologies. It contains various functional groups like phenolic hydroxyl, aliphatic hydroxyl, and carboxylic groups that can bind dye molecules through mechanisms like ion exchange or complex formation^12^. Therefore, lignin is a promising low-cost and eco-friendly adsorbent material for removing dyes from wastewater^8,9^. Despite the potential adsorption capabilities of lignin, pristine lignin (PL) exhibits relatively low pollutant removal compared to commercial absorbents. This is attributed to its structurally complex and heterogeneous three-dimensional architecture^13^. Alkali-extracted lignin^12^, organosolv lignin^14^, kraft lignin^15^, hydrolytic lignin^16^, and their modified versions have all been studied as adsorbents for cationic and anionic dyes like methylene blue, congo red, methyl orange, etc. Modification of lignin by esterification, sulfonation, amination, etc., or combination with other materials improves its dye adsorption capacity and selectivity significantly in many cases^17–22^. Metal ion doping, like iron, also enhances adsorption capacity^23^. The adsorption capacity ranged from around 20–120 mg/g for unmodified lignin types, and could reach above 200 mg/g when modified, like for aminated-lignin^17^. The adsorption process follows pseudo-second-order kinetics in most cases^13^, indicating a chemisorption mechanism involving sharing or exchanging electrons between dye molecules and lignin adsorbent. Equilibrium data fits the Langmuir model better than Freundlich in several cases, suggesting monolayer adsorption on a homogeneous surface^18^. Lignin-based adsorbents show rapid adsorption, reaching equilibrium in minutes to hours in most cases^16–19^. They also demonstrate good selectivity for cationic or anionic dyes in many single, binary, and multi-component dye systems^17,20–22^. Overall, lignin-based adsorbents offer a promising solution for removing methylene blue and other dyes from wastewater. However, they are cost-effective, abundant, and environmentally friendly. Further research and development are needed to optimize the adsorption capacity, selectivity, and regeneration of these materials to make them more practical for large-scale industrial applications.

In recent years, lignin nanoparticles (LNPs) have garnered significant interest for various applications due to their sustainable nature, high surface area, and tunable surface chemistry^14,24^. Several synthesis routes like solvent shifting, antisolvent precipitation, and hydrotropic treatment have been developed to produce lignin nanoparticles with controlled sizes and morphologies^11^. Using LNPs as adsorbents follows green chemistry principles by transforming abundant biopolymer waste streams into value-added nanomaterials, aligning with the goals of a sustainable circular bioeconomy^25,26^. The hydrotropic synthesis approach avoids toxic solvents and minimizes waste, addressing environmental concerns associated with conventional nanomaterial production^27^. Sustainable nanoparticle production avoids toxic reagents and minimizes waste compared to conventional methods^28^. The biopolymer feedstock, simple preparation, and pollutant remediation application align with sustainability targets for nanotechnology^29^. Continued efforts to implement green lignin valorization support circular bioeconomy transitions that are urgent for climate change mitigation.

While LNPs have been previously studied, a comprehensive understanding of structure-adsorbent property relationships is still lacking. This work provides new insights by systematically correlating the morphological and surface characteristics of LNPs produced via a hydrotropic method to their adsorption performance. The focus is on evaluating how nanoengineering affects pollutant removal capabilities through detailed isotherm and kinetic modeling analyses. The goal is to elucidate the mechanisms by which tailored synthesis strategies can optimize lignin nanomaterials as sustainable, effective adsorbents. This addresses the critical challenge of converting low-value lignin waste streams into higher-value-added nanoproducts with environmental applications. The hydrotropic nanoparticle preparation is also further optimized from previous protocols to achieve higher yield, better size control, and improved dispersion stability. The adsorbent testing examines a wider range of conditions and rigorous modeling compared to related works. By bridging the synthesis-structure–function relationships for LNPs as adsorbents, this work provides broader impacts on sustainable nanomaterial design and utilization of abundant biopolymer feedstocks.

## Materials and methods

### Materials

Softwood kraft lignin was obtained from Stora Enso to serve as the precursor for nanoparticle synthesis. Methylene blue dye (molecular weight: 319.85 g/mol; chemical formula: C_16_H_18_ClN_3_S) was acquired from Sigma Aldrich (United States) and used as a model adsorbate without further purification. The LNPs were synthesized using *p*-toluenesulfonic acid sodium salt (PTSA) obtained from Thermo Fisher Scientific Inc. (United States) as the hydrotropic agent (C_7_H_10_O_4_SNa, 213.190 g/mol, 98% purity).

### Synthesis of LNPs

The LNPs were prepared via a hydrotropic synthesis approach, employing PTSA as the hydrotrope. The synthesis procedure followed the method previously described by Cailotto et al.^11^. Briefly, 0.5 g of kraft lignin was added to 15 mL of 2 M PTSA solution and stirred for 1 h at 40 °C to allow lignin dissolution. The resulting black lignin-PTSA solution was filtered using Whatman No. 3 filter paper to remove undissolved particles. The filtrate was diluted with distilled water to reach 0.5 M PTSA concentration optimal for nanoparticle precipitation. The LNP dispersion was centrifuged at 21,130 × g (relative centrifugal force) for 20 min using a 15,000 rpm rotor, and the supernatant was separated. The purified LNP pellet was washed three times with distilled water by repeated centrifugation and decanting to remove residual PTSA salt. The nanoparticles were frozen at -40 °C and lyophilized overnight. The recovered lyophilized powder was resuspended in distilled water after 30 min of bath sonication and used for further characterization and adsorption experiments. The yield of LNPs obtained was determined gravimetrically based on triplicate synthesis batches.

### Characterizations of LNPs

For Fourier-transform infrared (FT-IR) analysis, the PL and LNP samples were prepared by mixing with potassium bromide (KBr) and pressing into transparent pellets. The PL powder was mixed with KBr at a ratio of 1:100 (w/w) before pressing. For the LNP sample, an aqueous dispersion of the nanoparticles was first lyophilized to obtain a dry powder, which was then mixed with KBr at a 1:100 ratio and pressed into pellets. The FT-IR spectra of the pellets were obtained in transmission mode over the wavenumber range 500 to 4000 cm^-1^.

The hydrodynamic particle size, polydispersity index (PDI), and zeta potential of the LNP suspensions were measured using the dynamic light scattering (DLS) technique on a HORIBA SZ-100 instrument (Horiba Scientific, Kyoto, Japan). The DLS measurements were performed at 25 °C using a He–Ne laser (632.8 nm) at a scattering angle 90°. The LNP suspensions were diluted to 1 mg/mL in distilled water and sonicated for 10 min before analysis to ensure good dispersion stability during the measurement. For the PL sample, the zeta potential measurement was more challenging due to its poor dispersibility and tendency to sediment rapidly in aqueous media. The PL powder was dispersed in distilled water at a concentration of 1 mg/mL by sonication for 10 min. The zeta potential measurement was performed immediately after sonication to minimize sedimentation effects. However, it should be noted that some degree of particle aggregation and sedimentation could still occur during the measurement, potentially affecting the accuracy of the reported zeta potential value. No additional electrolytes were added to the dispersions, so the ionic strength of the medium was determined solely by the presence of any dissociated ions or impurities in the distilled water used for dilution.

Atomic force microscopy (AFM) imaging of drop-cast LNP samples was done in tapping mode using an ENTEGRA probe microscope (NT-MDT Spectrum Instruments, Moscow, Russia). Silicon cantilevers with a force constant of 3.5–22.5 N/m and resonance frequency of 87–230 kHz were used.

The surface morphology of LNPs was examined by field-emission scanning electron microscopy (FE-SEM) using an MIRA3 LM microscope (TESCAN Orsay Holding, Brno, Czech Republic). Samples were sputter-coated with 5 nm gold and imaged at 15 kV accelerating voltage using secondary electron detection with three magnifications (35, 75, and 150 kx).

UV–visible spectroscopy for analyzing methylene blue concentrations was performed on a UV-1600 spectrophotometer (Shimadzu Corporation, Kyoto, Japan). Absorbance measurements were performed using 1 cm optical path length quartz cuvettes at the λmax of 664 nm for methylene blue.

The BET test was conducted using a Belsorp device to analyze the samples. Before the test, the samples underwent a vacuum treatment at 120 °C for 1 h to eliminate any moisture present in the samples. Subsequently, the dried samples were placed inside the BET test device to perform the analysis.

### Adsorption tests

The adsorption capacities of PL and LNPs for methylene blue were evaluated through batch experiments. A 200 mg/L methylene blue stock solution was prepared by dissolving 0.2 g of the dye in 1000 mL of deionized water. More dilute solutions (30, 50, 100 mg/L) were obtained by stepwise stock dilution. For the adsorption tests, 25 mg of adsorbent was added to 50 mL of methylene blue solution of specified concentration in a 100 mL glass beaker. The suspension was continuously stirred at 200 rpm using a magnetic stirrer, and sampling was performed over 1–100 min. The effects of solution pH (2, 7, 11) and adsorbent dosages (0.5, 1.0, 1.5, 2.0 mg/mL) were investigated. The amount of methylene blue adsorbed was calculated using UV–vis spectroscopy by measuring the supernatant absorbance at 664 nm and comparing it with a calibration curve. Methylene blue adsorption capacity (Q_t_) and removal efficiency (R) were quantified using the following equations:

1 $$Q_{t} = \left[ {\left( {C_{0} - C} \right) \times V} \right]/m$$

2 $$R = \left[ {\left( {C_{0} - C} \right)/C_{0} } \right] \times 100\%$$

where *q* is removal efficiency (%), *C*_*0*_ is the initial concentration, *C* is the methylene blue concentration in the solution at the time (t), Q_t_ is adsorption capacity (mg/g), V is solution volume, and *m* is the adsorbent mass.

Adsorption kinetics models, including pseudo-first-order and pseudo-second-order, were analyzed using the following equations:

3 $$ln \left( {Q_{e} - Q_{t} } \right) = lnQ_{e} - k_{1} t$$

4 $${\text{Q}}_{{\text{t}}} = {\text{ k}}_{2} Q_{e}^{2} {\text{t}}/\left( {1 + {\text{k}}_{2} {\text{Q}}_{{\text{e}}} {\text{t}}} \right)$$

where *Q*_*e*_ (mg/g) is the equilibrium adsorption capacity for methylene blue, *Q*_*t*_ (mg/g) is the adsorption capacity at time *t* (h) for methylene blue, *k*_*1*_ (1/min) is the pseudo-first-order rate constant, and *k*_*2*_ (g/mg h) is the pseudo-second-order rate constant. *Q*_*e*_ and *K*_*1*_ values can be obtained from the linear plot of *ln (Q*_*e*_*-Q*_*t*_*)* vs *t*.

Langmuir and Freundlich isotherm models were applied for the adsorption isotherms. *Q*_*e*_ vs *C*_*e*_ (residual MB supernatant concentration) plots were generated to evaluate the applicability of the Langmuir and Freundlich models. The Langmuir isotherm equation is:

5 $$\frac{1}{{Q_{e} }} = \frac{1}{{Q_{m} }} + \frac{1}{{\left( {K_{L} C_{e} Q_{m} } \right)}}$$

where *Q*_*m*_ is the maximum adsorption capacity (mg g^-1^), and *K*_*L*_ is the Langmuir constant related to adsorption energy. A linear *1/Q*_*e*_ vs *1/C*_*e*_ plot indicates Langmuir adsorption. The Freundlich isotherm is represented by:

6 $${\text{Log Q}}_{{\text{e}}} = {\text{log K}}_{{\text{F}}} + \frac{{\log C_{e} }}{n}$$

where *K*_*F*_ and *n* are Freundlich constants. A linear plot of log *Q*_*e*_ vs log *C*_*e*_ indicates a Freundlich adsorption mechanism.

## Results and discussion

### Characterizations

**Figure 1:**
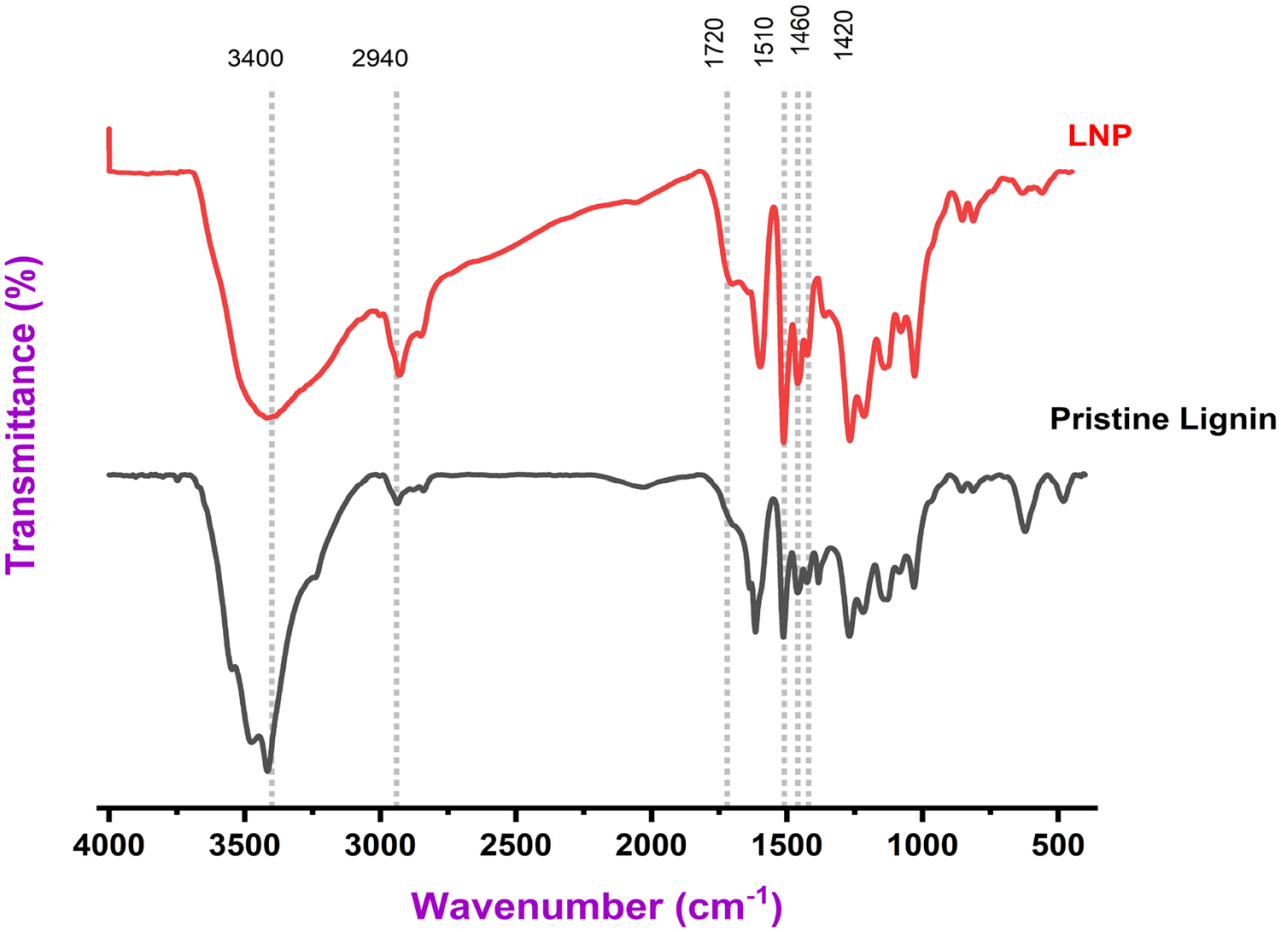
FT-IR analysis of functional groups in PL and LNPs.

The PL spectrum in Fig. 1 shows a broad band centered around 3400 cm^-1^, which can be attributed to O–H stretching vibrations in phenolic and aliphatic hydroxyl groups. This broad hydroxyl band is less prominent in the LNP spectrum, suggesting potential alterations to the hydroxyl content after hydrotropic synthesis. Further characterization such as ^31^P NMR would be needed to quantify differences in hydroxyl content between the samples. Both PL and LNP spectra exhibit a pronounced peak at 2940 cm^−1^, corresponding to C–H stretching in methyl and methylene groups. However, the intensity of this alkane C–H peak is greater for the LNP sample relative to the PL sample. The increased intensity of the 2940 cm^−1^ peak assigned to aliphatic C–H stretching modes in the LNP spectrum compared to PL indicates enrichment of methyl and methylene groups in the nanoparticles relative to the bulk lignin. This enhancement could arise from several factors during the hydrotropic synthesis process:

1. Preferential self-assembly and precipitation of lignin fractions richer in aliphatic/alkyl side chains to form the nanoparticle core, leading to their relative enrichment compared to the bulk lignin.
2. Potential cleavage of aryl ether linkages and exposure of aliphatic segments in lignin macromolecules under acidic hydrotropic conditions, increasing the proportion of methylene groups.
3. Changes in the molecular environment/conformation of alkyl side chains in the nanoparticle assembly that could amplify the intensity of their vibrational modes.

To confirm the origin and quantify any changes, complementary analytical techniques like quantitative ^13^C NMR or alkane carbon analysis would be beneficial. However, the FT-IR data provides an initial indication of potential structural modifications influencing the aliphatic composition when lignin transitions from bulk to nanoparticle form via hydrotropic synthesis. A prominent band arising from aromatic skeleton vibrations of lignin's phenylpropane units appears at 1510 cm^−1^ in both spectra. This aromatic skeletal vibration peak occurs at a slightly lower wavenumber for LNPs than PL. Further structural analysis such as HSQC NMR would be needed to determine if this shift indicates modifications of the aromatic rings during nanoparticle synthesis, as speculated by Cailotto et al.^11^. The peaks centered around 1460 cm^−1^ and 1420 cm^−1^ associated with C-H deformations are conserved in both PL and LNP but with subtle shifts to lower wavenumbers for the LNP sample. The FT-IR analysis revealed a new carbonyl C=O stretching peak at 1720 cm^−1^ for the LNPs synthesized via the hydrotropic method, which was absent in the spectrum of the PL. The origin of these carbonyl functionalities cannot be definitively assigned from the current FT-IR data alone. However, several plausible explanations arise from insights provided by previous studies on lignin nanoparticle synthesis and characterization. Cailotto et al.^11^ reported that hydrotropic treatment of lignin under acidic conditions can induce certain oxidation reactions, leading to the formation of new carbonyl/carboxyl groups. Similarly, Richter et al.^25^ observed the appearance of carbonyl bands in FT-IR spectra of lignin nanofibers produced using an oxidative system with TEMPO (2,2,6,6-tetramethylpiperidine-1-oxyl radical) and sodium hypochlorite. They attributed this to the oxidative cleavage of Cα-Cβ bonds in lignin's propyl side chains. Therefore, mild oxidation of some lignin subunits under the acidic hydrotropic conditions (*p*-toluenesulfonic acid at 40 °C) employed in this study could potentially explain the emergence of carbonyl groups in the LNP spectra.

Table 1 presents the characterization data on particle size and surface charge for PL and LNPs synthesized by a hydrotropic method. The yield of LNPs produced via the hydrotropic technique is reported as 74% based on the oven-dried weight of lignin recovered after the synthesis. This moderate yield indicates that not all the dissolved lignin participates in nanoparticle precipitation, as a small portion remains insoluble after filtration, and a fraction stays solubilized in the hydrotrope solution rather than precipitating out to form the LNPs. Measurement of the z-average hydrodynamic diameter by DLS shows that PL forms large aggregates of around 6899 nm (6.8 µm) when dispersed in aqueous medium, with a PDI of 1.56, indicating a highly heterogeneous and broadly distributed system. For the LNP sample, the average hydrodynamic diameter was 208 nm, with a PDI of 0.14, suggesting a relatively narrow and uniform particle size distribution. However, it should be noted that the use of DLS for measuring particle sizes in this range (> 1–2 μm) is subject to significant limitations and potential inaccuracies due to violations of the underlying assumptions of the technique, such as the Rayleigh approximation, single scattering events, and the influence of slow Brownian motion and sedimentation effects on larger particles or aggregates. Therefore, the reported value of 6899 nm should be interpreted with caution, as it may not accurately represent the true size distribution of the PL aggregates in aqueous media. Complementary techniques more suitable for larger particle sizes, such as static light scattering, laser diffraction, or microscopy-based image analysis, could provide more reliable size characterization of the PL aggregates. In contrast, the LNPs exhibit a significantly reduced average particle size of 208 nm, confirming the capability of the hydrotropic method to facilitate precipitation and self-assembly of dissolved lignin fractions into nanoparticles. Zeta potential measurements reveal the highly anionic nature of PL surfaces in water, with a value of -66 mV. However, despite this high negative charge, PL exhibits extensive aggregation when dispersed in aqueous medium, with particles sizes around 6900 nm as measured by DLS. The aggregation likely arises from intermolecular interactions between the complex lignin polymer chains overcoming the electrostatic repulsion.

**Table 1:** Particle size and surface charge analysis of PL and LNP.

Absorbent	Average zeta potential charge (mV)	Average particle size (nm)	Yield (%)
PL	−66 ± −4.5	6899 ± 215	–
LNP	−13.3 ± −2.1	208 ± 18	74*

*Based on oven-dried weight.

**Figure 2:**
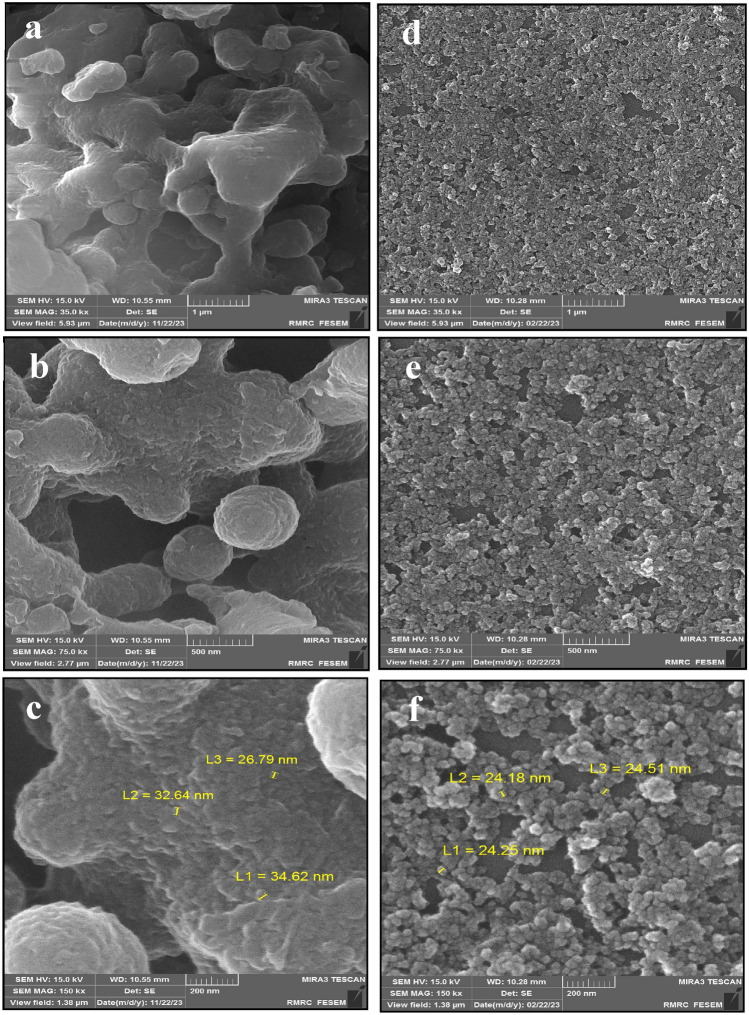
SEM analysis of micropore formation in PL (**a**, **b**, and **c**) versus LNP (**d**, **e**, and **f**) with different magnifications.

The SEM micrographs at lower magnification (Fig. 2a,d) reveal that PL has an irregular, heterogeneous morphology consisting of variably sized and shaped particulate aggregates up to several microns. This random structure arises from the complex, amorphous nature of lignin polymer chains. In contrast, the LNPs exhibit consistent spherical shapes and more uniform size distribution on the order of 100–300 nm diameter. This confirms hydrotropic treatment effectively breaks down the large, irregular particles in PL into smaller, more regular nanoparticles. Further magnification to 150,000X (Fig. 2b,e) shows the PL particle surfaces display a smooth, non-porous exterior morphology without a distinguishable fine structure. This reflects the amorphous polymer properties. However, abundant micropores are visible, covering the entire exterior of the LNPs. The evenly dispersed pores have diameters around a few hundred nanometers. They likely form as packing defects during nanoparticle assembly under hydrotropic conditions. The highest magnification micrographs (Fig. 2c,f) show a featureless exterior for the PL particles. Meanwhile, the extensive macroporosity of the LNPs can still be distinctly observed. The pores exhibit partially ordered arrangement and uniform ~ 100–200 nm dimensions.

**Figure 3:**
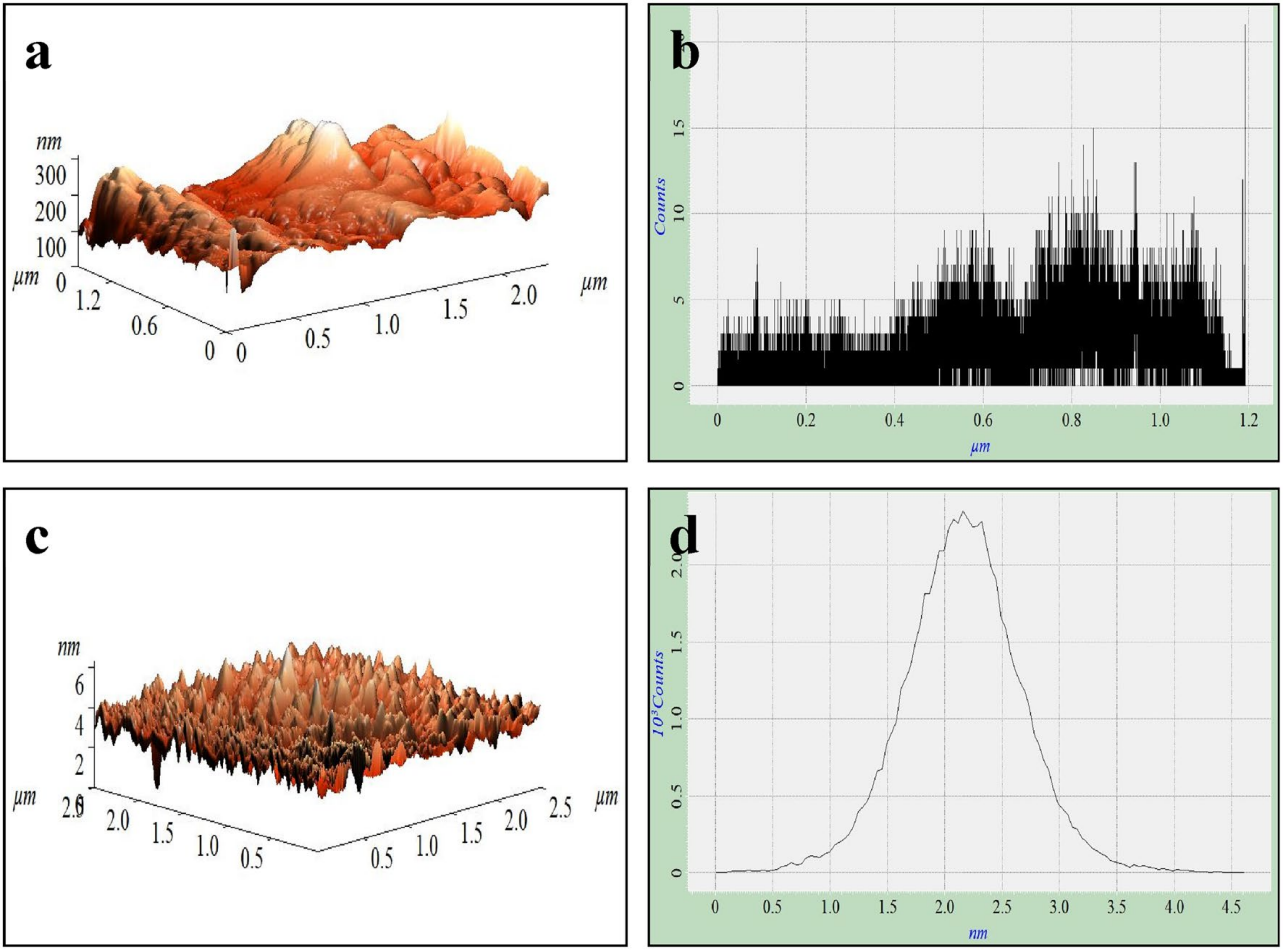
AFM imaging of (**a**, **b**) PL versus (**c**, **d**) dispersed LNPs.

Figure 3 presents AFM characterization of the morphology and topography of PL compared to LNPs. The AFM images reveal key differences between the materials. PL exhibits an irregular, heterogeneous surface structure consisting of large aggregated particles up to 500 nm in size. This extensive agglomeration likely arises from strong intermolecular interactions between the complex lignin polymer chains that resist dispersion. Despite the highly anionic nature of PL surfaces in water, as revealed by the zeta potential measurement of -66 mV, PL exhibits extensive aggregation when dispersed in an aqueous medium, with particle sizes around 6900 nm as measured by DLS. This aggregation behavior can be attributed to strong intermolecular interactions between the complex lignin polymer chains, which overcome the electrostatic repulsion forces arising from the negative surface charge. In contrast, the LNPs display a much smoother and uniform morphology composed of well-dispersed, spherical nanoparticles around 100–200 nm in diameter. The narrower particle size distribution and reduced agglomeration verify that the hydrotropic synthesis successfully broke down the bulk lignin polymer into discrete nanoparticles. Quantitative roughness analysis indicates PL has a very high surface roughness exceeding 50 nm, while LNPs possess much lower roughness around 20 nm. It is to be noted that the roughness values quantify the degree of unevenness of the external surface morphology. The root-mean-square (RMS) roughness values are 48.3 nm and 11.2 nm for PL and LNPs, respectively. The significantly decreased surface roughness of nanoparticles can be attributed to their regular, spherical shape and morphology compared to the amorphous, irregular structure of PL. These AFM findings on nanoparticle size, dispersion, and roughness agree well with results from previous studies. For example, Cailotto et al.^11^ reported successful fabrication of uniform LNPs around 100 nm diameter with smooth morphology using a similar hydrotropic approach. The reduced roughness was proposed to enhance dispersion stability. Overall, the AFM characterization verifies that the hydrotropic method produces well-controlled LNPs with reduced aggregation and improved dispersion stability compared to PL. This agrees with prior works demonstrating the capability of hydrolytic techniques to nanoengineer biomass-derived polymers into tunable nanomaterials^30–32^. The tailored nanoparticle properties impart enhanced adsorbent functionality.

The hydrodynamic diameter and zeta potential of the LNP dispersions were characterized before and after the addition of methylene blue dye using DLS. The LNP dispersions at a concentration of 1 g/L were used for the initial zeta potential measurement before dye addition, exhibiting an average diameter of 208 nm and a zeta potential of -13.3 mV (Table 1). To analyze the effect of methylene blue adsorption, 200 mg/L of the dye was introduced to the 1 g/L LNP dispersion, resulting in a final methylene blue:LNP ratio of 1:5 by weight. After methylene blue addition and adsorption, the average particle size increased to 292 nm, indicating some aggregation occurred upon binding of the positively charged methylene blue cations onto the negatively charged LNPs, which reduced the electrostatic repulsion. Concurrently, the zeta potential became more negative, shifting to -22.1 mV. The more negative zeta potential value corresponds to a greater negative surface charge on the LNPs from the adsorbed cationic methylene blue molecules.

Table 2 presents the specific surface area (SSA), total pore volume, and average pore diameter results obtained from BET analysis of PL and LNPs. The BET test was conducted to characterize the porosity and surface area, influencing adsorption capacity and kinetics. Before testing, the samples were pretreated by vacuum drying at 120 °C for 1 h to remove moisture. The dried samples were then analyzed using a Belsorp instrument to obtain the nitrogen adsorption–desorption isotherms for BET calculations. The results show that LNPs have a fourfold higher SSA of 14.9 m^2^/g compared to only 3.4 m^2^/g for PL. The increased surface area of nanoparticles arises from their smaller size and porous structure produced during hydrotropic synthesis. Total pore volume, representing the cumulative volume of micropores and mesopores, is 0.12 cm^3^/g for LNPs versus 0.013 cm^3^/g for PL. The one-order of magnitude higher pore volume of nanoparticles is attributed to the introduction of macroporosity during the self-assembly of lignin fractions under hydrotropic conditions. The average pore diameter calculated using the adsorption data is approximately double for LNPs (32.4 nm) to PL (15.8 nm). The larger pore sizes facilitate rapid diffusion and transport of adsorbate molecules to interior surfaces. It is to be noted that the average pore sizes obtained from gas adsorption represent the internal microporosity. Thus, the AFM and BET characterization techniques provide complementary structural insights into the external roughness versus internal porosity of the materials. The lower surface roughness for LNPs arises from their smooth, spherical nanoparticle morphology; whereas greater internal porosity originates from packing voids between particles.

**Table 2:** Surface area and porosity characterization of lignin materials by BET analysis.

Sample	SSA (m^2^/g)	Total pore volume (cm^3^/g)	Average pore diameter (nm)
PL	3.4	0.01342	15.8
LNP	14.9	0.1203	32.4

### Adsorption

Table 3 reports the equilibrium adsorption capacity (Q_e_) and pseudo-second-order kinetic rate constant (k_2_) values obtained from batch adsorption experiments for different initial methylene blue concentrations. For PL, the Q_e_ is low, ranging from 20.58 to 20.74 mg/g, and shows minimal variation with increasing initial dye concentration from 30 to 200 mg/L. This indicates that the adsorption sites of PL become saturated even at low methylene blue concentrations. In contrast, the LNPs exhibit a steady increase in Q_e_ from 54.91 to 127.91 mg/g as the initial concentration rises from 30 to 200 mg/L. The higher Q_e_ confirms the greater adsorption capacity of LNPs owing to their porous structure and high surface area, allowing more dye to be adsorbed before saturation of the available sites.

**Table 3:** Quantitative comparison of methylene blue adsorption kinetics.

Sample	C0 (mg/L)	Qe (mg/g)	k2 (mg/g.min)
PL	30	20.58	0.02436
	50	20.59	0.07125
	100	20.68	0.022173
	200	20.74	0.008926
LNP	30	54.91	1.438056
	50	92.68	0.032825
	100	122.83	0.021924
	200	127.91	0.016538

**Figure 4:**
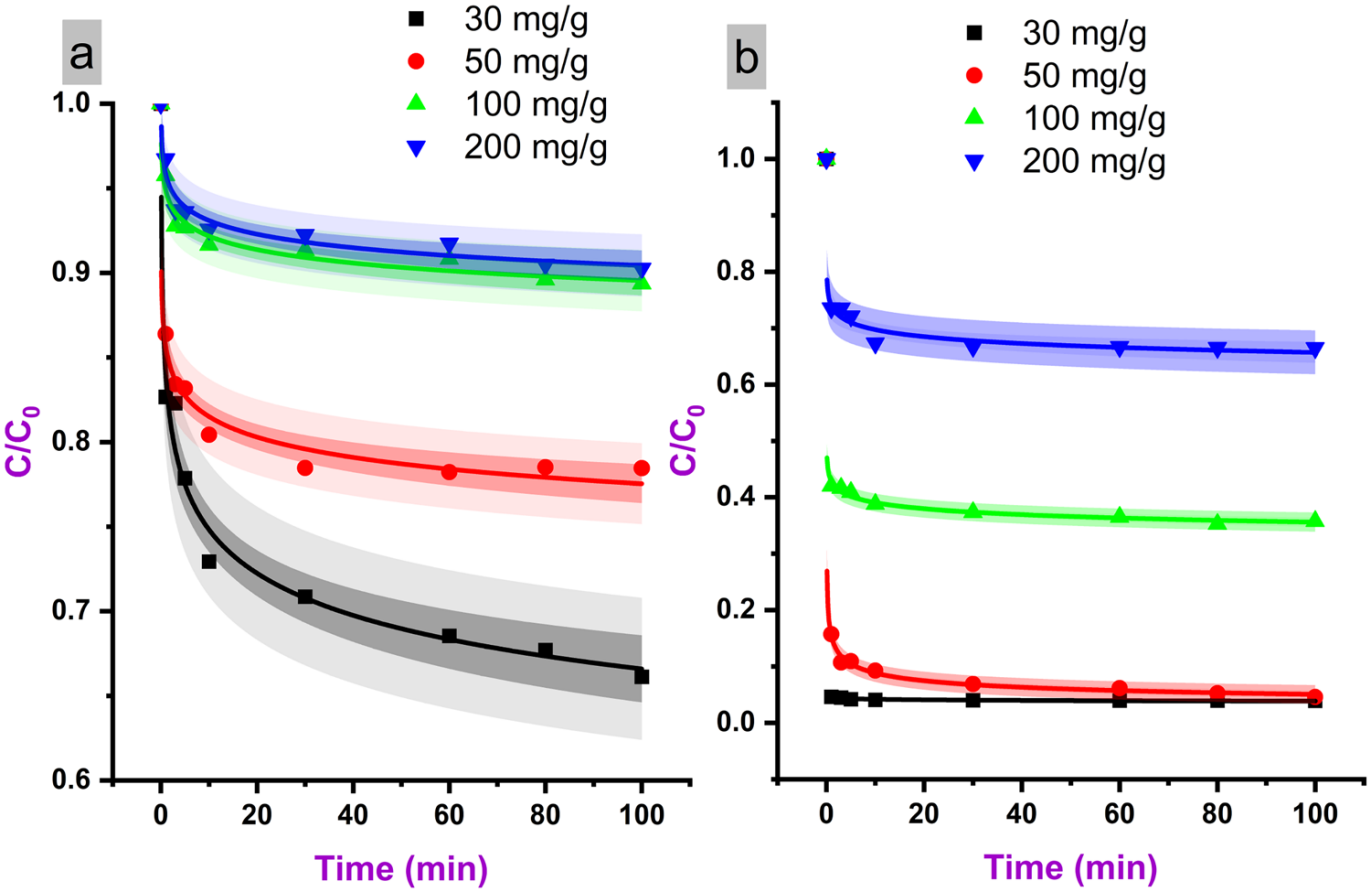
Adsorption efficiency profiles showing methylene blue uptake by LNPs.

The k_2_ constant represents the speed of absorption, with higher values indicating faster kinetics. The k_2_ for LNPs is 1–2 orders of magnitude higher than PL, reflecting significantly quicker absorption. This rapid absorption rate is also clearly evidenced in the absorption efficiency versus time plots shown in Fig. 4. Equilibrium absorption for LNPs is attained within 3 min, while PL requires around 10 min to reach equilibrium. The ultrafast absorption displayed by LNPs can be attributed to the facile diffusion of methylene blue through the micropores to access interior binding sites. Additionally, the numerous surface functional groups of the nanoparticles promote rapid chemisorption. In contrast, slow pore diffusion and fewer available sites limit the absorption rate for PL.

**Figure 5:**
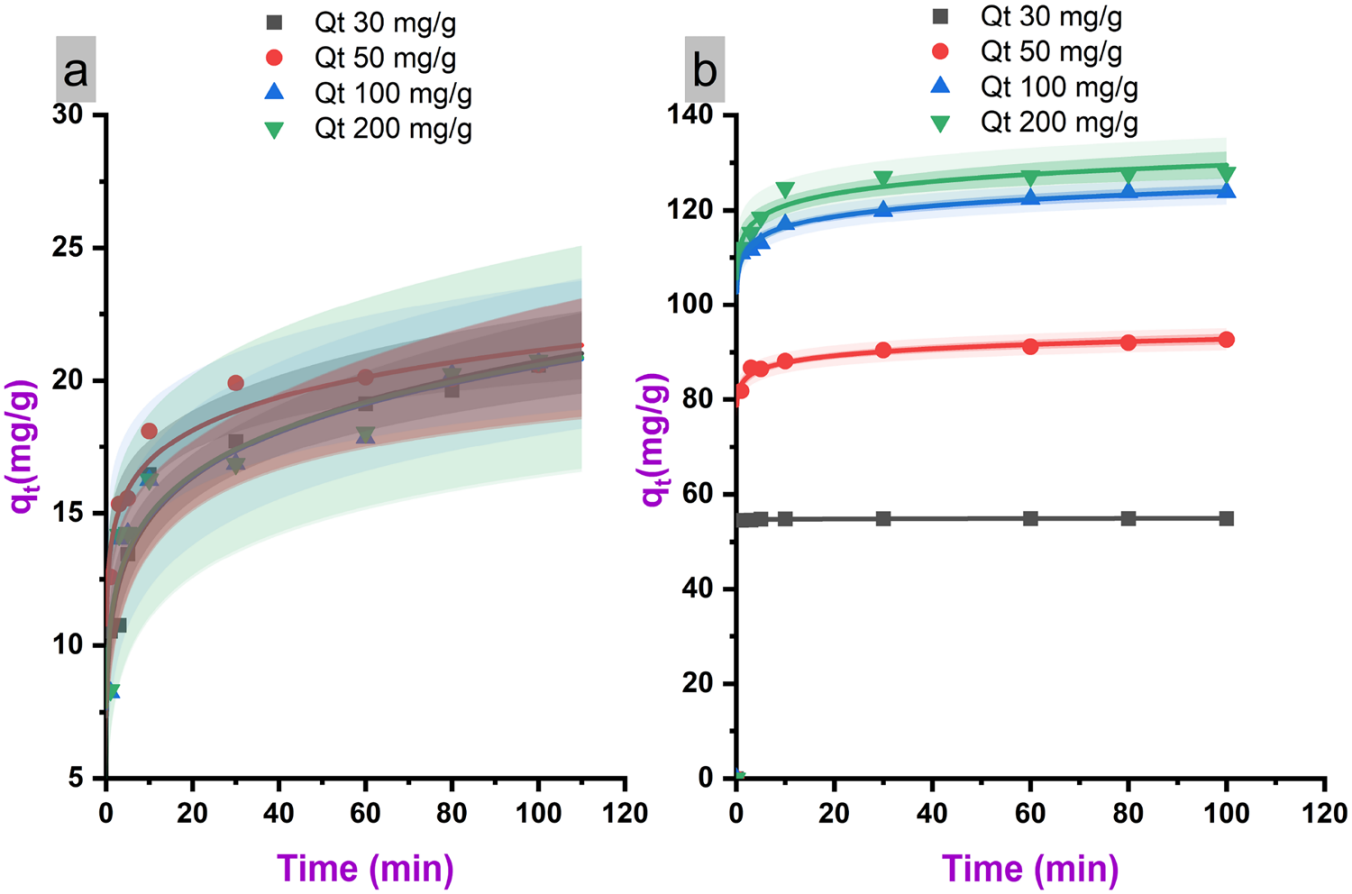
Adsorption capacity behavior of PL (**a**) and LNPs (**b**) across a range of methylene blue concentrations.

Figure 5 presents adsorption capacity versus time profiles for LNPs at different initial concentrations of methylene blue from 30 to 200 mg/L. In all cases, the adsorption capacity increases sharply within the first minute of contact time, indicating ultrafast adsorption kinetics. Equilibrium is attained rapidly within 3 min for the LNPs, after which no significant change in adsorption capacity is observed up to 100 min. The rapid attainment of equilibrium adsorption, irrespective of dye concentration, highlights the excellent mass transfer and accessibility of the binding sites within the macro- and mesoporous LNPs.

The initial adsorption rate and equilibrium adsorption capacity are both found to increase with higher initial methylene blue concentration. For the LNPs, the initial adsorption rate is 54.91 mg/g/min at 30 mg/L, rising to 127.91 mg/g/min at 200 mg/L. The final equilibrium values follow the same trend, with capacities of 62.57, 96.15, 132.46, and 146.82 mg/g obtained for the 30, 50, 100, and 200 mg/L initial concentrations, respectively. The increase can be attributed to a greater driving force for mass transfer and higher occupancy of active binding sites when higher dye concentrations are present initially. However, the incremental enhancements in rate and capacity diminish as the concentration rises. This indicates gradual saturation of the available adsorption sites. The plateauing of capacities suggests that the maximum adsorption capacity is approached by 200 mg/L, though this was not determined experimentally.

Overall, Fig. 5 demonstrates that both the kinetics and capacity of LNPs for methylene blue adsorption are concentration-dependent. The ultrafast adsorption behavior is maintained over the range of environmentally relevant concentrations examined. This illustrates the potential for efficiently removing methylene blue across a wide range of wastewater compositions using the LNPs. The results complement the kinetic data presented in Table 3 to quantify the superior adsorption performance of LNPs compared to PL.

**Figure 6:**
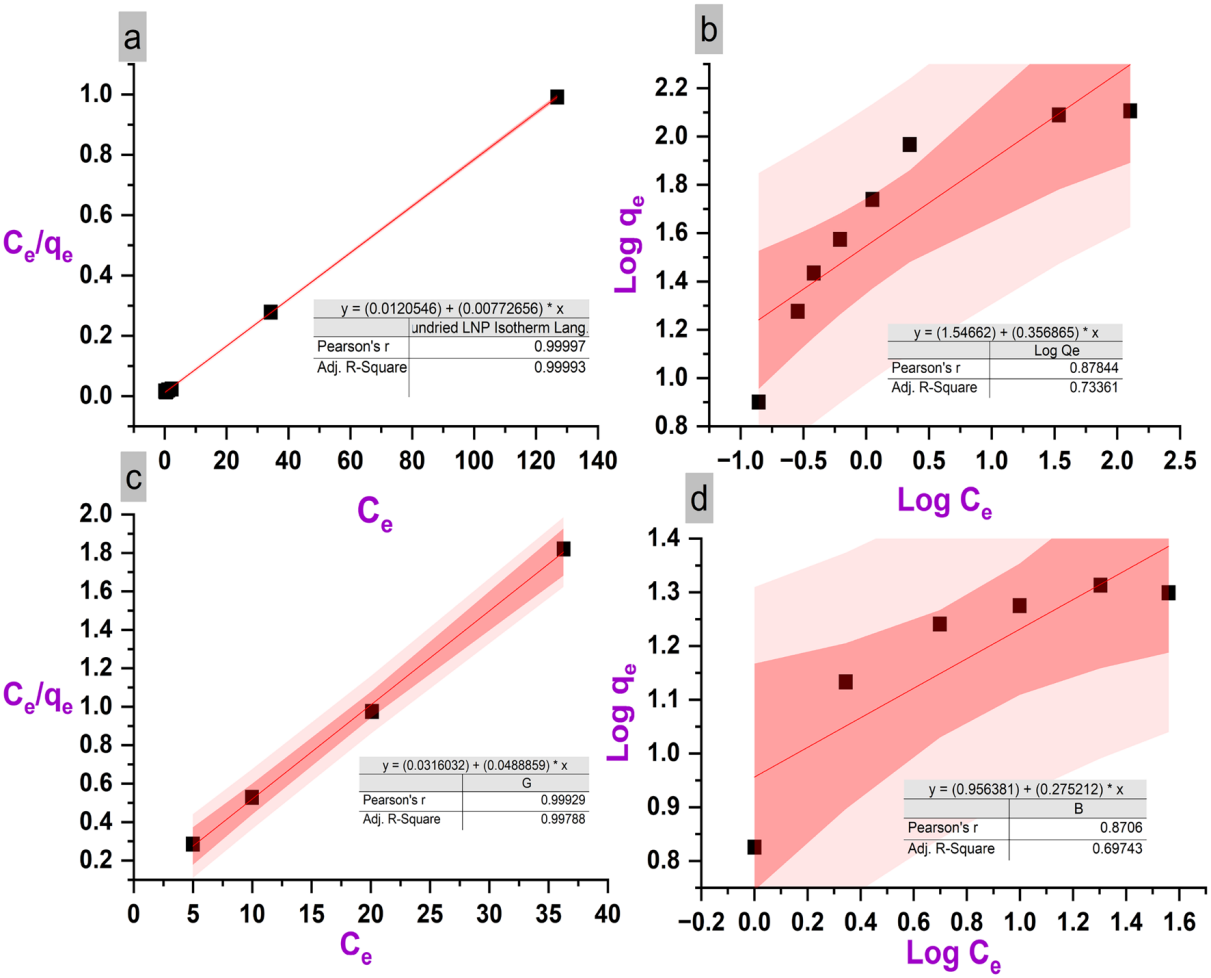
Comparison of Langmuir (**a**, **c**) and Freundlich (**b**, **d**) isotherm models for methylene blue adsorption onto LNPs (**a**, **b**) and PL (**c**, **d**).

Figure 6 shows the Langmuir and Freundlich isotherm fits for the experimental equilibrium adsorption capacity data. The Langmuir model assumes monolayer adsorption onto a surface with homogeneous binding sites, while the Freundlich model describes multi-layer adsorption on a heterogeneous surface. For both LNPs and PL, the Langmuir model shows a better linear fit with higher R^2^ values of 0.977 and 0.974, respectively. The conformity to the Langmuir isotherm suggests that methylene blue adsorption occurs via monolayer coverage on the relatively uniform surface functional groups of the lignin materials. From the Langmuir modeling, maximum adsorption capacities (Q_m_) of 334.4 and 24.16 mg/g are obtained for LNPs and PL, respectively. The 14-fold higher Q_m_ value quantitatively confirms the substantially greater methylene blue adsorption capacity of LNPs compared to the pristine bulk lignin. The Langmuir model implies monolayer adsorbate coverage governs the overall adsorption process. However, some surface heterogeneity is evident from the minor applicability of the Freundlich model. The multilayer adsorption contribution may occur at very high concentrations near saturation. Nonetheless, the predominance of Langmuir behavior verifies that the uniform macro- and mesoporous structure of the LNPs primarily facilitates monolayer adsorption, giving rise to their higher methylene blue adsorption capacity.

Table 4 presents the isotherm modeling results of methylene blue adsorption onto PL and LNPs. The Langmuir and Freundlich isotherm models were applied to analyze the equilibrium adsorption data. For both LNPs and PL, higher R^2^ values were obtained using the Langmuir model (0.977 and 0.974) compared to the Freundlich model (0.733 and 0.690). This indicates the Langmuir isotherm provides a better fit to the data, suggesting monolayer adsorption onto relatively uniform lignin surface sites is the primary mechanism governing methylene blue uptake. The maximum adsorption capacity (Q_m_) calculated from the Langmuir model was 334.4 mg/g for LNPs versus only 24.16 mg/g for PL. The substantially higher Q_m_ of nanoparticles reflects their greater methylene blue adsorption capacity resulting from the high surface area and porous structure providing increased active binding sites. The Langmuir equilibrium constant K_L_ was lower for LNPs (0.185 L/mg) than PL (0.398 L/mg), indicating a stronger binding affinity of dye molecules to the PL surface. Overall, the equilibrium modeling results verify Langmuir monolayer adsorption as the predominant mechanism, with LNPs exhibiting significantly higher methylene blue adsorption capacity than PL owing to their favorable porous structure and surface chemistry.

**Table 4:** Adsorption isotherm model parameters for methylene blue uptake on PL and LNPs.

Isotherm models	PL	LNP
Langmuir		
K_L_ (L/mg)	0.398	0.185
R^2^	0.974	0.977
q_e_ (mg/g)	24.166	334.4
Freundlich		
K_F_ ((mg/g)/L mg)^1/n^)	9.04	35.206
R^2^	0.69	0.733
l/n	0.275	0.356

**Figure 7:**
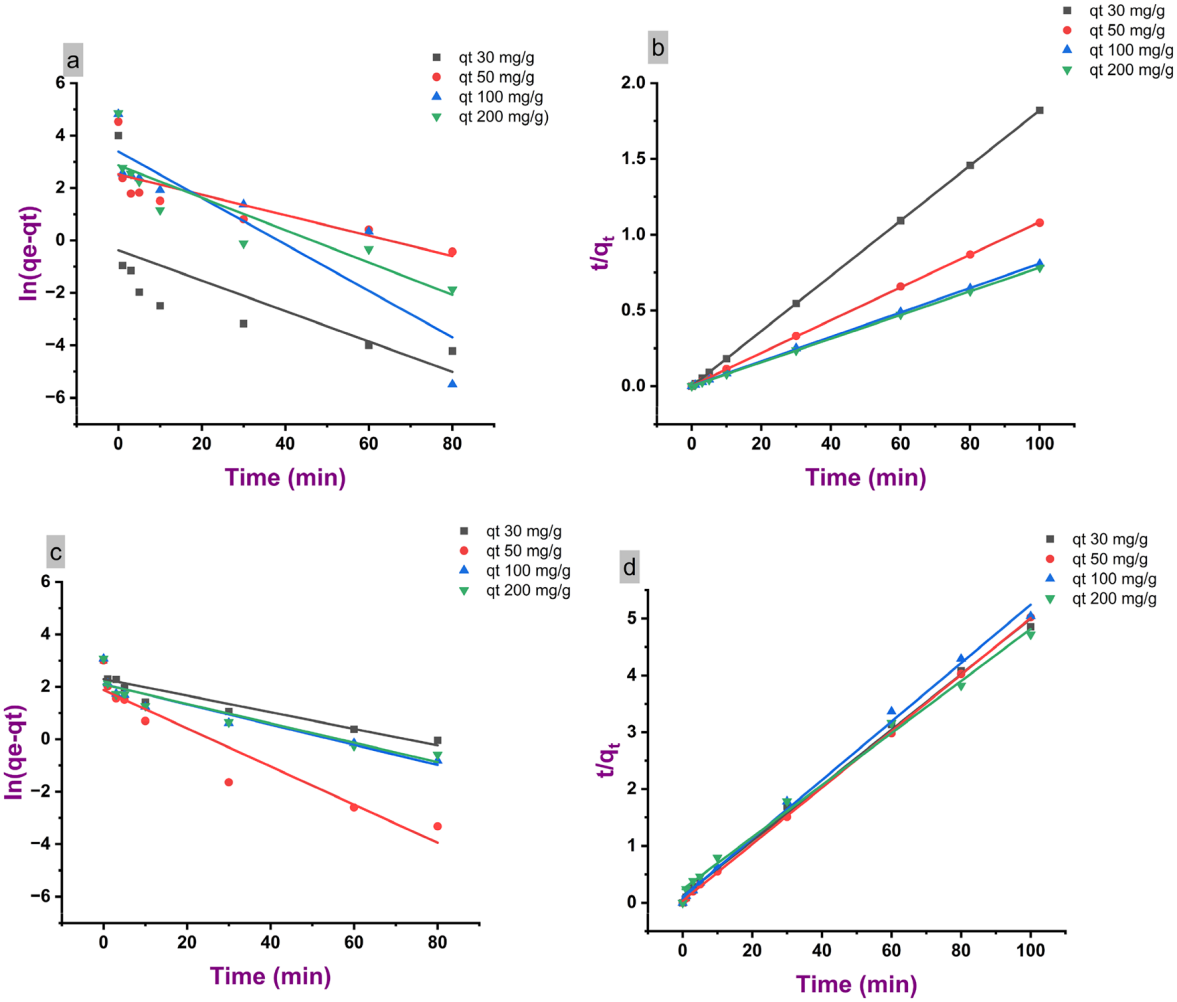
Pseudo-first-order (**a**, **c**) and pseudo-second-order (**b**, **d**) kinetic modeling of methylene blue adsorption on PL (**a**, **b**) and LNPs (**c**, **d**).

Figure 7 compares the pseudo-first order and pseudo-second order kinetic models applied to the adsorption of methylene blue onto PL and LNPs. The pseudo-first order model assumes that adsorption occurs by diffusion, while the pseudo-second-order model implies that the adsorptive-adsorbent interaction process is the rate determining step. For both LNPs and PL, the pseudo-second-order model provided a better fit to the experimental data, with higher coefficient of determination (R^2^) values of 0.99 compared to 0.33–0.40 for the pseudo-first-order model. This suggests that the adsorptive-adsorbent interaction is the step controlling the adsorption kinetics. The improved applicability of the pseudo-second-order model can be attributed to the abundant surface functional groups on lignin that can interact with methylene blue molecules. Additionally, the pseudo-second-order rate constant (k^2^) was over an order of magnitude higher for LNPs (0.03 g/mg·h) versus PL (0.07 g/mg·h). The faster kinetics of LNPs arises from the nanoparticle morphology providing greater accessibility to the chemically active binding sites. Overall, the kinetic modeling verifies that the interaction between methylene blue and lignin governs the adsorption, with LNPs exhibiting superior performance to PL.

Figure 7 compares the pseudo-first-order and pseudo-second-order kinetic models applied to the adsorption of methylene blue onto PL and LNPs. The pseudo-first-order model assumes adsorption occurs by diffusion and physical interactions, while the pseudo-second-order model involves chemisorption via electron exchange or sharing between adsorbent and adsorbate. For both LNPs and PL, the pseudo-second-order model (Fig. 7b,d) provided a better fit to the experimental data, with higher coefficient of determination (R^2^) values of 0.99 compared to 0.33–0.40 for the pseudo-first-order model (Fig. 7a,c). This suggests chemisorption is the predominant mechanism controlling the adsorption kinetics. The improved applicability of the pseudo-second-order model can be attributed to the abundant surface functional groups on lignin that can participate in chemisorptive interactions with methylene blue molecules. Additionally, the pseudo-second-order rate constant (k^2^) was 0.07 g/mg·h for PL versus 0.03 g/mg·h for LNPs. The higher k^2^ of PL reflects its faster adsorption kinetics arising from greater accessibility of methylene blue molecules to the chemically active binding sites. In general, the kinetic modeling confirms that the adsorption process is primarily governed by the interaction between methylene blue and lignin. Notably, the performance of LNPs surpasses that of PL adsorption efficiency.

Table 5 presents the kinetic parameters obtained from fitting the pseudo-first-order and pseudo-second-order models to the adsorption data of methylene blue onto PL and LNPs. The applicability of the models was evaluated based on the coefficient of determination (R^2^) values. For both LNPs and PL, the pseudo-second-order model provided a better fit to the experimental data, with higher R^2^ values of 0.99 compared to 0.33–0.40 for the pseudo-first-order model. This confirms the adsorptive-adsorbent interaction is the rate-determining step controlling the adsorption kinetics. The pseudo-second-order rate constant (k_2_) was 0.07 g/mg·h for PL versus 0.03 g/mg·h for LNPs. The faster kinetics of PL arises from the nanoparticle morphology of LNPs providing greater accessibility to the chemically active binding sites. It is to be noted that the superior fit of the pseudo-second-order kinetic model over the pseudo-first-order model suggests that chemisorption governs the rate-limiting step during methylene blue adsorption onto the lignin materials^33^. This chemisorption mechanism has been widely reported for dye adsorption on lignin-based adsorbents, attributed to the interactions between the dye molecules and the phenolic/ionic functional groups on the lignin surface^34^.

**Table 5:** Kinetic modeling parameters for methylene blue adsorption on PL and LNPs.

Adsorbate	Pseudo-first-order		Pseudo-second-order	
	k_1_ (mg/g h)	R^2^	k_2_ (g/mg h)	R^2^
PL	−0.0006793	0.33	0.07125	0.99
LNP	−0.00028	0.40	0.032825	0.99

## Conclusions

This study demonstrated the enhanced adsorption capabilities of LNPs produced through a hydrotropic method compared to PL powder. Multiple characterization techniques verified the successful fabrication of spherical LNPs with ~ 200 nm diameter using the hydrotropic approach. Batch adsorption experiments exhibited up to 14 times higher dye removal capacity and ultrafast equilibrium uptake under 3 min for LNPs over PL. Kinetic modeling revealed the interaction between methylene blue and lignin phenolic functional group as the primary mechanism, with LNPs displaying faster absorption rates from greater accessibility to active binding sites. Isotherm analysis indicated predominant Langmuir monolayer coverage on relatively uniform lignin nanoparticle surface sites. In summary, the hydrotropic treatment produced high-capacity LNPs and rapid pollutant removal from their tailored structure and surface chemistry. The maximum adsorption capacity of 334.4 mg/g for methylene blue is moderately higher than typical values reported for unmodified lignin adsorbents.

Using LPNs as adsorbents follows green chemistry principles by transforming abundant waste streams into value-added nanomaterials. Sustainable nanoparticle production avoids toxic reagents and minimizes waste compared to conventional methods. Hydrotropic self-assembly requires only water and an organic hydrotrope salt, facilitating benign processing and recycling. The biopolymer feedstock, simple preparation, and pollutant remediation application align with sustainability targets for nanotechnology. Mainly, replacing less eco-friendly adsorbents tackles key challenges of materials specificity and safety identified to harmonize nano innovation with environmental objectives.

## Data Availability

Data will be made available on request.
